# Testicular Assessment in a Selected Group of Young Buffaloes Using Doppler Ultrasonography

**DOI:** 10.1002/vms3.70672

**Published:** 2025-12-26

**Authors:** Rodrigo dos Santos Albuquerque, Janaina Barroso dos Santos, Moises Moreira Lima, Aluizio Otavio Almeida da Silva, Kayan da Cunha Rossy, Michel Santos e Cunha, Gabriela Melo Alves dos Santos, Francisco Décio de Oliveira Monteiro, Luisa Pucci Bueno Borges, Maria Eduarda Bastos Andrade Moutinho da Conceição, Bruno Moura Monteiro, Rinaldo Batista Viana, Leandro Nassar Coutinho, Moysés dos Santos Miranda, Pedro Paulo Maia Teixeira

**Affiliations:** ^1^ Veterinary Medicine Institute Federal University of Pará Castanhal Pará Brazil; ^2^ Federal Institute of Tocantins (IFTO) Campus Araguatins Araguatins Tocantins Brazil; ^3^ Department of Veterinary Clinic and Surgery State University of São Paulo Jaboticabal São Paulo Brazil; ^4^ Institute of Health and Animal Production Federal Rural University of the Amazon Belém Pará Brazil

**Keywords:** andrology, buffalo bulls, buffalo in puberty, testicular haemodynamics, testicular ultrasound

## Abstract

Doppler ultrasonography is a valuable tool for assessing testicular vascular function, but its application in buffaloes, particularly during puberty, remains limited. Establishing reference haemodynamic parameters for young bulls is crucial for early sire selection and reproductive management. The purpose of the study was to evaluate testicular haemodynamics and parenchymal echotexture in Murrah bulls of the pubertal‐stage and Mediterranean buffalo bulls using adapted bovine Doppler ultrasonography protocols. Thirteen clinically healthy buffalo bulls (7 Murrah, 6 Mediterranean) between 12 and 18 months old were examined. Testicular biometric data, greyscale echotexture (ImageJ) and Doppler indices, peak systolic velocity (PSV), end diastolic velocity (EDV), pulsatility index (PI), and resistive index (RI) were recorded for the supratesticular artery. PSV was significantly higher in 16–18‐month‐old bulls compared to those aged 12–15 months (*p* < 0.05), suggesting increased arterial perfusion at the onset of puberty. Other Doppler indices did not show significant age‐related variation. B‐mode imaging revealed homogeneous echotexture in younger bulls, while older bulls exhibited more prominent hyperechoic linear patterns, consistent with the progress of spermatogenesis. Only the supratesticular artery was consistently visualised; intratesticular and marginal arteries were not measurable due to anatomical constraints. In conclusion, the peak systolic velocity (PSV) increased significantly with age, suggesting its potential as an early indicator of pubertal onset and breeding soundness. In contrast, other Doppler indices (PI, RI, EDV) remained stable, reflecting consistent vascular resistance during this developmental phase. B‐mode ultrasonography revealed age‐related echotextural changes, with the appearance of hyperechoic lines in older bulls likely associated with advancing spermatogenesis. Among testicular vessels, only the supratesticular artery was consistently visualised, the intratesticular and marginal arteries were not detected. By adapting bovine Doppler protocols to buffaloes, this study addresses a gap in veterinary andrology and provides a standardised approach for future research.

## Graphical abstract

Doppler ultrasonography revealed increased peak systolic velocity (PSV) in pubertal buffalo bulls (16–18 months), indicating enhanced arterial perfusion, while other hemodynamic indices remained stable. Age‐related echotextural changes in testicular parenchyma were observed, supporting PSV as an early marker for pubertal onset and breeding soundness in buffalo andrology.

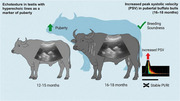

## 1 Introduction

Doppler ultrasonography has become an indispensable tool for evaluating testicular vascular function and predicting reproductive potential in bovine andrology (Velasco and Ruiz 2021). Although its use is well established for identifying subfertility in young bulls, its use in buffalo reproduction remains limited despite the economic value of the species and the unique reproductive physiology of this species. Although recent studies have shown links between testicular vascular patterns and semen quality in buffalo, data on pubertal animals are lacking, a critical gap given physiological differences from cattle (Gloria et al. 2018; Albuquerque et al. 2023). Puberty in buffaloes involves distinct anatomical and functional changes compared to cattle, and these changes can directly influence the soundness of the breeding.

In this context, our study was designed to evaluate testicular haemodynamics and echotexture in 12–18‐month‐old Murrah and Mediterranean buffalo bulls using Doppler ultrasonography. Our findings complement existing research on buffalo reproduction and may help identify early markers of reproductive competence (Sharma et al. 2022). This study adapts bovine Doppler techniques to address a key limitation in buffalo andrology (Rossi et al. 2025). Our results may support research seeking to identify high‐potential sires before sexual maturity, thereby optimising genetic progress and economic returns.

Doppler analysis provides valuable information on vascular perfusion, blood flow characteristics and spermatogenic activity (Souza et al. 2014), complementing the structural information obtained from conventional B‐mode ultrasonography. It helps diagnose vascular disorders and assess semen quality (Ortiz‐Rodriguez et al. 2017). Although widely used in human testicular evaluation (Schurich et al. 2009) and equine reproduction (Ortega‐Ferrusola et al. 2014; Pozor and McDonnell 2004), applications in buffalo are emerging. Recent work has confirmed its value for monitoring seasonal haemodynamics and semen quality (Samir et al. 2023) and advancing andrological techniques (Samir et al. 2021), however, most of the work focusses on adult animals, leaving a gap in characterisation of vascular development during puberty.

The study provides useful basic data on the testes of a selected group of young buffaloes from the Murrah and Mediterranean breed buffaloes that can support the identification of infertile bulls due to abnormal testicles, addressing the gaps identified in recent work on seasonal vascular dynamics (Samir et al. 2023) and application of Doppler in animal andrology (Samir et al. 2021; Abdelnaby 2022), generating foundational data that can guide future research, support the early diagnosis of subfertility and contribute to the refinement of andrological evaluation protocols in buffalo breeding systems.

Therefore, this study aimed to assess testicular haemodynamics and parenchymal echotexture in a selected group of young buffalo bulls of Murrah and Mediterranean breeds using Doppler ultrasonography, establishing basic data for the andrological evaluation of animals.

## 2 Materials and Methods

### 2.1 Ethics Approval and Experimental Design

This study was carried out according to the recommendations of the National Council for Experimentation Control in Brazil (CONCEA). This research was approved by the Animal Ethics and Welfare Committee of the Federal University of Pará (protocol N ° 9963300120).

In this investigation, a total of 13 buffaloes were used, under the same environmental, nutritional and sanitary conditions (Table 1).

**TABLE 1:** Characteristics of the Murrah and Mediterranean buffaloes in the study: Age ranges, weight, health status and feeding regimen.

ID	Breeds	Age groups	Peso (kg)	Health status	Feeding	Supplementation	Water availability
1	Murrah	12–15	386	All clinically healthy	Elephant grass (*Pennisetum purpureum, Schum*) pasture + balanced concentrate feed	Mineral supplementation	Ad libitum
2	Murrah	12–15	347				
3	Murrah	12–15	328				
4	Murrah	12–15	328				
5	Murrah	16–18	495				
6	Murrah	16–18	419				
7	Murrah	16–18	407				
8	Mediterranean	12–15	355				
9	Mediterranean	12–15	347				
10	Mediterranean	12–15	328				
11	Mediterranean	16–18	491				
12	Mediterranean	16–18	429				
13	Mediterranean	16–18	410				

The experiment was conducted at the Animal Reproduction Biotechnology Center (CEBRAN), Federal University of Pará, located in the municipality of Castanhal, Pará, Brazil (lat 1°18′17.9″ S and long 47°56′ 30.2″ W).

### 2.2 Clinical Examination and Testicular Biometrics

Physical examination consisted of a clinical evaluation of the external and internal structures of the reproductive system by palpation of the testicles, epididymis and spermatic cord, in addition to the accessory sexual glands and the preputial region of the animals. After examining the symmetry and shape of the testicles, epididymis and structures of the pampiniform plexus, biometric measurements of testicular size were performed with the aid of a pachymeter and measuring tape, according to the methodology of the Brazilian College of Animal Reproduction (Brazilian College of Animal Reproduction 2013).

The testicular volume was determined through the mathematical model suggested by Unanian et al. (2000) with the mathematical equation of the cylinder volume (VOLC), VOLC = [width (LARG) / 2]^2^ × Π × length (COMP), π = 3.141592654, and another one proposed by Bailey et al. (1998), the spherical prolate equation, testicular volume (VOLP) = 2 × [4/3 × Π × {width (LARG) / 2}^2^ × [length (COMP)/2)]. In addition, the volume of each testicle was obtained using the average of the two mathematical formulas and the total testicular volume obtained by adding the volume of the right and left testicles, expressed in mm^3^ (Cunha et al. 2019; Teixeira et al. 2012).

### 2.3 Echogenicity Analysis

The greyscale values of the ultrasound images of the testicular parenchyma were expressed in units of pixel intensity defined by numerical values ranging from 0 to 255, with 0 representing dark echogenicity (anechoic) and 255 light echogenicity (hyperechoic). The testicular echogenicity (ECHOt) of the images was expressed in numbers of pixels/area using ImageJ software, based on an image processing program developed by the National Institutes of Health in the United States. In addition, the program calculates the area and generates pixels in user‐defined values (Schneider 2012).

The average numbers of pixels units for each testes were obtained in four areas measuring approximately 1 square centimetre randomly selected in the longitudinal and transverse sections of the testicular parenchyma. Then, the ECHOt of the general average was determined for each animal using these average values (Brito et al. 2012; Pinho et al. 2013).

The images were classified from 0 to 2 according to the visual scale of testicular ultrasound assessment, where 0 corresponds to a homogeneous image without changes in echogenicity, 1 to a homogeneous image with minor changes in echogenicity, and 2 to a classification of heterogeneous images with hypoechoic and hyperechoic areas. In addition, animals classified between 0 and 1 were categorised by image examination as animals without alterations. During ultrasound examination, animals classified as 2 on the visual scale were categorised as animals with evidenced alterations.

### 2.4 B‐Mode and Doppler Ultrasound

For ultrasound evaluation, a MyLabTM 30Vet Esaote model device (Rapallo, GE, Italy) was used. The buffaloes were taken to the containment trunk and a B‐mode ultrasound evaluation of the testicular stroma was performed, using an acoustic gel on the scrotum on the side of the right and left testicles, according to the methodology defined by Teixeira et al. (2012), in the sagittal, transversal and frontal planes. To avoid discrepancies, all ultrasound procedures were conducted by the same operator.

The testicles were evaluated using colour mapping to study the vascular architecture. The device was calibrated with a gain of between 52 and 62 and a pulse repetition frequency of between 6.6 and 8 MHz using a linear transducer. To identify blood flow in the supratesticular artery, intratesticular artery and capsular artery, B‐mode and colour Doppler were performed. Subsequently, spectral Doppler was applied to quantify the speed of blood flow within the vessel. During the procedure, the three regions of the artery were examined to assess whether Doppler evaluation was necessary, and the examination was performed at the evaluated sites.

**FIGURE 1:**
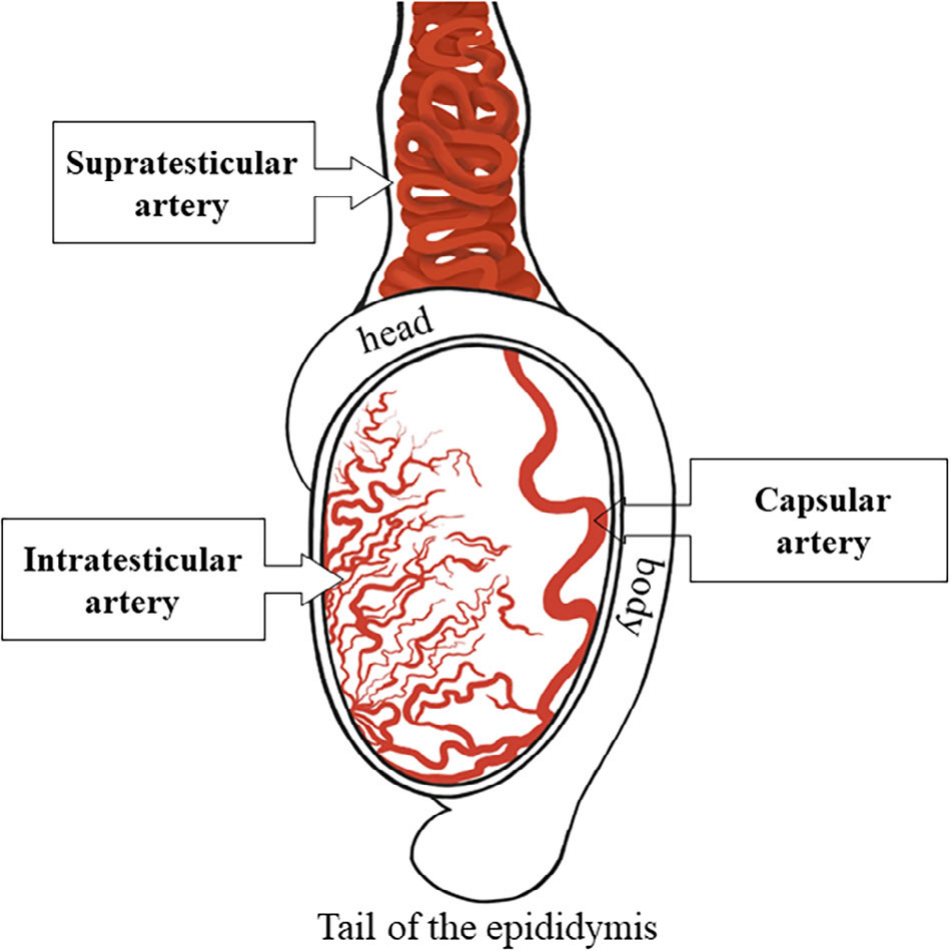
Vascular architecture of the buffalo testicle, illustrating three different locations of the branches of the testicular artery guided by a transducer and the degree of tortuosity, convolutions and caliber of the vessels at the evaluation points during the ultrasound examination in Doppler mode.

For Dopplerfluxometric evaluation of the supratesticular artery region, the transducer was positioned cross‐sectionally at the insertion of the scrotum to the proximal region of the testis, in the region of the pampiniform plexus. In an effort to locate the intratesticular artery, the transducer was positioned transversely and longitudinally at the centre of each testis. To assess the capsular artery, the same procedure was performed by lateralising the transducer in both testicles toward the marginal region (Figure 1). This imaging modality was used to recognise the different anatomical structures of the testicles and diagnose potential disorders.

Flowmeter Doppler mean and standard deviation data was measured, as well as their relationship to animal testicular data. The calculated Doppler parameters were the resistivity index (IR) and pulsatility index (PI), peak systolic velocity (PSV) and end‐diastolic velocity (EDV).

### 2.5 Statistical Analysis

Data on testicular biometrics, ECHOt and Dopplerfluxometric indices were submitted to the mean ± standard deviation. A contrast was carried out between the mean values obtained for echogenicity and Dopplerfluxometric indices for each group using the two‐way analysis of variance test (ANOVA). Moreover, significance was set at 5% (*p* < 0.05). The evaluation points of the analysed arteries were classified in frequency according to the possibility of measuring the parameters in Doppler mode. The classification of the testicular visual ultrasound scale was also evaluated in frequency, with (2) or without changes (0 and 1) verified by B‐mode ultrasound images. All data were analysed using the BioEstat 5.3 statistical package.

The statistical methodology used in this study was selected based on established practices in veterinary andrology and reproductive biometry. Descriptive statistics (mean ± standard deviation) were used for continuous variables, an appropriate measure for normally distributed biometric and haemodynamic data (Velasco and Ruiz 2021; Gloria et al. 2018). ANOVA was applied to assess the effects of breed and age group on Doppler indices and echogenicity, a validated method for evaluating interactive effects in pubertal development studies (Albuquerque et al. 2023). Frequency analysis was utilised for categorical data (e.g., ultrasound scores), a conventional approach for such outcomes (Sharma et al. 2022). The significance threshold was set at *p* < 0.05, a standard alpha level adopted in biomedical research to balance error rates (Rossi et al. 2025; Souza et al. 2014). All analyses were performed using the BioEstat 5.3 statistical package (Ortiz‐Rodriguez et al. 2017).

## 3 Results

The results of testicular biometrics were processed as well as echogenicity on a pixel scale, and the Dopplerfluxometric indices found in young buffalo can be seen in Table 2. With the exception of PSV, which was higher in animals aged 16–18 months, there was no significant difference between the parameters analysed in animals aged 12–15 months and in buffaloes aged 16–18 months.

**TABLE 2:** Values of scrotal perimeter, total testicular volume, testicular ultrasound econdensity and Dopplerfluxometric parameters measured in the supratesticular artery in the sperm cord of buffalo aged 12–15 months (*n* = 7) and 16–18 months (*n* = 6) in breeding centre.

		Parameters					
Age group	SP (cm)	TTV (mm^3^)	ECHOt (pixels)	PI	RI	PSV (cm/s)	EDV (cm/s)
12 a 15 months of age	27.42 ± 1.81	351.42 ± 142.06	92.18 ± 15.67	1.80 ± 0.38	0.80 ± 0.08	5.19 ± 1.18^a^	2.00 ± 0.85
16 a 18 months of age	29.08 ± 1.31	392.93 ± 99.11	102.25 ± 14.68	1.57 ± 0.99	0.72 ± 0.18	7.00 ± 1.59^b^	3.25 ± 1.58
*p*‐value	0.09	0.56	0.25	0.57	0.30	0.03	0.09

*Note*: Data show values expressed as mean ± standard deviation.

Different letters in the same column indicate different values (*p* < 0.05).

Abbreviations: ECHOt, testicular echogenicity; EDV, end diastolic velocity; IR, resistivity index; PI, pulsatility index; PSV, peak systolic velocity; SP, scrotal perimeter; TTV, total testicular volume.

**FIGURE 2:**
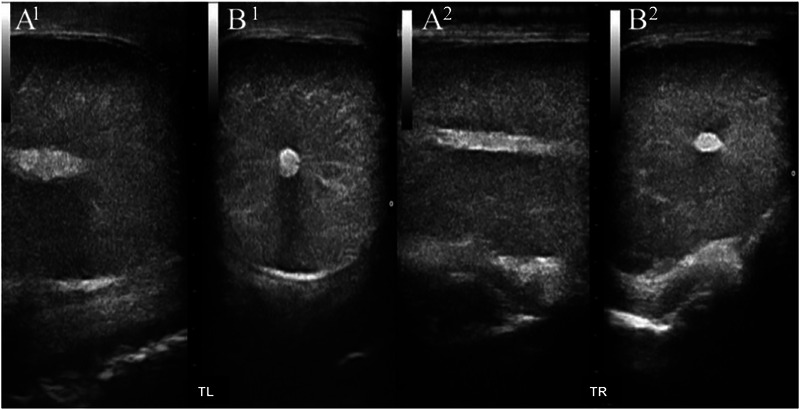
B‐mode ultrasound of testicular parenchyma in buffalo bulls (12–15 months), demonstrating homogeneous echotexture distributed along the parenchyma of the left (LT) and right (RT) testicles in the longitudinal (A) and transverse (B) planes. Epididymal structures were not captured in this view. LT: left testis; RT: right testis.

The echogenicity and echotexture of the animals showed a homogeneous pattern with slightly tortuous hyperechoic linear regions distributed centrifugally along the testicular parenchyma toward the mediastinum to the external region of the testis, as shown by B‐mode ultrasonography. The mediastinum region was easily seen as a hyperechoic point in a transverse plane and as a central hyperechoic line in a longitudinal plane (Figure 2).

**FIGURE 3:**
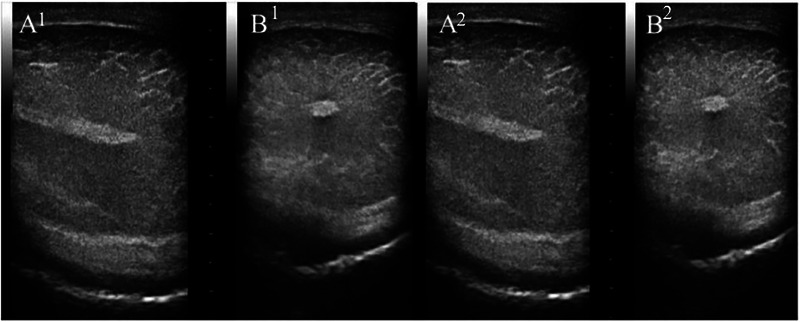
B‐mode ultrasound image showing more intense hyperechoic lines (“visual scale of testicular ultrasound Type 1), distributed along the left (LT) and right (RT) testicular parenchyma in the longitudinal (A) and transverse (B) planes found in four aged buffaloes between 16–18 months in a breeding centre (Castanhal, Pará, Brazil).

Among the 13 animals examined during the B‐mode ultrasound examination, four buffaloes between the ages of 16 and 18 months had more intense hyperechoic lines along the testicular parenchyma, the visual scale of the testicular ultrasound was Type 1 (Figure 3). Compared to the other two animals of 16 and 18 months and seven animals between the ages of 12 and 15 months that had a homogeneous pattern without the marked presence of these streaks, visual scale of testicular ultrasound Type 0. However, all animals (100%) were classified without changes according to the visual testicular ultrasound scale (Type 0 and 1).

The supratesticular artery was easily seen in all animals (100%), but the intratesticular artery or the capsular artery in the marginal region of the testicles could not be evaluated.

**FIGURE 4:**
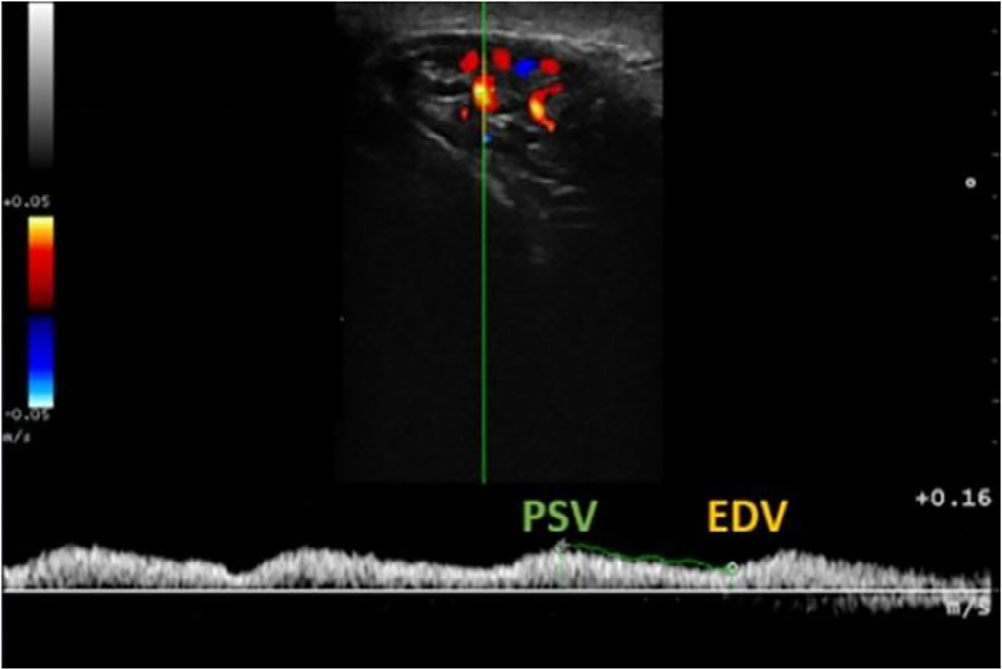
Triplex Doppler ultrasound image demonstrating the arterial tracing and coloured mapping of the vascularization of the testicular artery and its branches with blood flow in different shades, revealing the variation in blood velocity within the vessel located in the spermatic cord. The vertical green caliper line refers to the position of the supratesticular artery located in the central area of the blood vessel to determine the spectral curve during systole (PSV) and diastole (EDV). Flow Velocity Integral (FVI) 3.6 cm, Pulsatility Index (PI) o.68, Resistive Index (RI) 0.50, Peak Systolic Velocity (PSV/Vp) 7.1 cm/s, End‐Diastolic Velocity (EDV) 3.5 cm/s, Time‐Averaged Mean Velocity (Vmn) 5.2 cm/s.

In the testicular artery of the spermatic cord, colour Doppler ultrasonography revealed a tortuous pattern along its path, and spectral Doppler showed a monophasic pattern of pulsatile wave with low vascular resistance and discrete PSV and long EDV (Figure 4).

## 4 Discussion

This study focused on young buffalo bulls (12–18 months) to establish baseline Doppler parameters during the critical pre‐pubertal (12–15 months) and early pubertal (16–18 months) stages, as these ages represent key developmental transitions in buffalo testicular function (Samir et al. 2023; Abdelnaby 2022). While human Doppler applications prioritize disease diagnosis, veterinary andrology requires developmental benchmarks for breeding soundness evaluation, particularly in buffalo where puberty onset (∼15–18 months) precedes proven fertility by 6–12 months. Our group stratification was based on: (1) Documented testicular echotexture during the onset of buffalo spermatogenesis initiation (Albuquerque et al. 2023), and (2) the need to identify subfertility markers before sexual maturity (Gloria et al. 2018).

The data specifically aim to support early bull selection by correlating Doppler indices (PSV, PI) with parenchymal changes, with future studies planned to validate these parameters against seminal quality in mature bulls. This foundational work bridges the critical gap between diagnostic and developmental applications of testicular Doppler in buffalo andrology. The present study describes the Dopplerfluxometric indices of the testicular artery in the selected group of young buffalo. Although there are anatomical and functional variations between the different species, the reference data from other species was used to compare the Dopplerfluxometric characteristics of buffaloes and other animals due to the scarcity of research on buffaloes.

Colour Doppler ultrasonography provides critical functional insights that conventional B‐mode imaging cannot capture, particularly to assess testicular vascular dynamics in buffalo bulls. Although B‐mode ultrasound effectively evaluates structural parameters such as testicular volume and echotexture (Bailey et al. 1998), colour Doppler enables real‐time visualization of haemodynamic changes associated with pubertal development and spermatogenic activity (Samir et al. 2021). In buffalo, this technology proves especially valuable given the vascular architecture of the testicular vascular system of the species (Sharma et al. 2022), where conventional ultrasound often fails to detect early perfusion abnormalities preceding morphological changes (Albuquerque et al. 2023).

The ability of the technique to quantify PSV and resistive index (RI) provides objective markers of testicular function, as demonstrated by significant increases PSV in 16–18‐month‐old buffalo bulls during the onset of puberty (Abdelnaby 2022). These haemodynamic parameters are more strongly correlated with future semen quality than B‐mode measurements alone (Gloria et al. 2018), offering a 3–4‐month predictive advantage for breeding soundness evaluations (Pozor and McDonnell 2004). Furthermore, colour Doppler detects subtle vascular changes during seasonal fertility fluctuations (Samir et al. 2023) and hormonal treatments (Ortiz‐Rodriguez et al. 2017), making it indispensable for comprehensive buffalo andrology programs where conventional ultrasound provides only anatomical snapshots.

The significant increase in PSV observed in buffalo bulls aged 16–18 months compared to younger animals (12–15 months) likely reflects the onset of puberty, which typically occurs between 15–18 months in Murrah buffalo (Abdelnaby 2022). This haemodynamic change aligns with established testicular development patterns, where higher testosterone levels during puberty enhance arterial vasodilation and systolic flow velocity (Samir et al. 2023). The absence of significant differences in other Doppler indices (PI, RI, EDV) suggests that while systolic perfusion increases, overall vascular resistance remains stable during this transition period, a phenomenon also documented in pubertal bulls (Gloria et al. 2018).

The findings correlate with histological changes in the testicular parenchyma of buffalo, where advancing spermatogenesis (evidenced by hyperechoic lines in older bulls) increases metabolic demand without substantially altering the microvascular architecture (Albuquerque et al. 2023). The results complement reproductive biometry studies showing that testicular volume and diameter of the seminiferous tubule undergo more pronounced changes after 18 months (Rossi et al. 2025), explaining why PSV (a sensitive marker of early vascular adaptation) was the only parameter demonstrating significant divergence between age groups. This haemodynamic pattern mirrors observations in stallions, where elevations of PSV precede seminal quality improvements by 2–3 months (Pozor and McDonnell 2004), supporting the utility of PSV as an early indicator of pubertal progression in buffalo andrology.

Six animals between the ages of 16 and 18 months showed a degree of heterogeneity in the testicular parenchyma, as evidenced by pixel analysis and visual scale. This can be correlated with intense histomorphological changes that occur during puberty, such as the differentiation of seminiferous tubules in the presence of mature germ cells that reported higher numerical values of pixels in animals starting the spermatogenesis process in a study conducted in sheep (Bartlewski et al. 2017). A previous study carried out in sheep using ultrasound, histology and immunohistochemistry showed strong correlations between testicular echotexture and diameter of the seminiferous tubules during the first wave of spermatogenesis (Giffin and Bartlewski 2014).

Analysis of velocimetric indices can also be a valuable method for monitoring vascular changes during puberty in several species. In prepubertal dogs, Dopplerfluxometric indices were lower relative to animals after puberty, an unusual finding when you consider that sexual maturity promotes vasodilation and increased blood flow, if there is a vasodilation PI and RI will decrease. However, the authors attributed these differences to the length, thickness and elasticity of the wall of the vessels that influenced the calculation of the IR, in addition to the use of animals of various breeds that may have caused changes in haemodynamics (Souza et al. 2015).

The PSV and EDV values for buffaloes were characterised and a monophasic wave pattern was determined with a slight systolic peak and low diastolic flow but with accelerations due to distal vasodilation in the spermatic cord. The same is observed in reports described by Zelli et al. (2013) who identified that dogs have characteristics of a vessel with low resistance with slow systolic flow followed by long accelerated diastolic flow, referring to the interpretation that these velocity patterns are from animals without any type of haemodynamic change.

In stallions, Pozor and McDonnell (2004) evaluated the waveform of the cardiac cycle of the testicular artery at the localisation of the spermatic cord, observing the biphasic wave pattern with significant differences in blood flow between systolic and diastolic velocities. In terms of analysis in cattle, the supratesticular artery blood flow waveforms also revealed a monophasic pattern with a very evident systolic and diastolic peak in healthy animals of various breeds (Barca Junior et al. 2018).

In all of the studies mentioned above, the species submitted for haemodynamic evaluations by Doppler ultrasonography presented a tortuous pattern of the testicular artery in the region of the spermatic cord that was easily observed during examination. The research conducted in stallions (Pozor and McDonnell 2004) and cattle (Gloria et al. 2018) assessed the path of the testicular artery in three different locations: In the spermatic cord, in the testicles region and in the intratesticular region, finding blood flow and cardiac cycle formation in all regions analysed, which could not be verified in the present study; however, waves were formed in the pampiniform plexus, making it difficult to perform intratesticular evaluations in the base of the mediastinum and marginal regions close to the gonadal wall.

This fact may be related to the anatomy of buffaloes, which in turn are characterised by presenting smaller structures of the reproductive system, including testicles and the vessels that promote irrigation. In buffaloes, the marginal part of the testicular artery is very long relative to the position and size of the testicles and has a greater degree of tortuosity compared to other species such as horses, camelids and rabbits. Sheep have a very similar anatomy to buffaloes, with much more tortuous branches of the vessels (Elayat et al. 2014).

Elbaz et al. (2019) reported that the branches of the intratesticular artery were visualized in the testicular parenchyma; however, they had small points of colour that were insufficient to generate flow analysis in this region. Unlike what Gloria et al. (2018) described in cattle, the parameters of vascular perfusion can be measured in the supratesticular artery, testicular artery in the marginal portion and branches of the intratesticular artery.

The pattern of vascular architecture in buffaloes is more complex due to the large number of convoluted branched vessels present both in the spermatic cord and in the gonad (Elayat et al. 2014). This reduction in blood flow velocity and difficulty in visualisation using colour Doppler restricts the flow Doppler analysis only in the supratesticular artery in the pampiniform plexus along the spermatic cord.

Therefore, the use of Doppler ultrasound to assess the haemodynamics of the testicular artery is feasible in buffaloes and can only be performed in the region of the branches of the supratesticular artery in the spermatic cord. Animals in the experimental group aged between 12 and 15 months showed a more homogeneous echogenicity pattern, while buffaloes aged between 16 and 18 months showed marked hyperechoic lines along the testicular parenchyma that can be considered normal for the species.

## 5 Conclusions

This study offers valuable information on testicular haemodynamics and parenchymal echotexture in young buffalo bulls using Doppler ultrasonography. The PSV increased significantly with age, suggesting its potential as an early indicator of pubertal onset and breeding soundness. In contrast, other Doppler indices (PI, RI, EDV) remained stable, reflecting consistent vascular resistance during this developmental phase. B‐mode ultrasonography revealed age‐related echotextural changes, with the appearance of hyperechoic lines in older bulls likely associated with advancing spermatogenesis. Among testicular vessels, only the supratesticular artery was consistently visualised; intratesticular and marginal arteries were not detected. By adapting bovine Doppler protocols to buffaloes, this study addresses a gap in veterinary andrology and provides a standardised approach for future research.

## Author Contributions

Conceptualisation; methodology; project administration and supervision: Luisa Pucci Bueno Borges, Bruno Moura Monteiro, Rinaldo Batista Viana, Leandro Nassar Coutinho and Pedro Paulo Maia Teixeira. Data curation; formal analysis and investigation and resources: Janaina Barroso dos Santos, Moises Moreira Lima, Aluizio Otavio Almeida da Silva, Kayan da Cunha Rossy, Michel Santos e Cunha and Gabriela Melo Alves dos Santos. Visualisation, writing—original draft and writing—review and editing: Rodrigo dos Santos Albuquerque, Moysés dos Santos Miranda, Gabriela Jaques Rodrigues, Maria Eduarda Bastos Andrade Moutinho da Conceição, Francisco Décio de Oliveira Monteiro and Pedro Paulo Maia Teixeira.

## Funding

The authors have nothing to report.

## Ethics Statement

This study was carried out in accordance with the recommendations of the National Council for Experimentation Control in Brazil (CONCEA). This research was approved by the Animal Ethics and Welfare Committee of the Federal University of Pará (protocol N ° 9963300120).

## Conflicts of Interest

The authors declare no conflicts of interest.

## Data Availability

All data generated or analysed during this study are included in this published article.
